# Mechanochemical subcellular-element model of crawling cells

**DOI:** 10.3389/fcell.2022.1046053

**Published:** 2022-12-05

**Authors:** Mitsusuke Tarama, Kenji Mori, Ryoichi Yamamoto

**Affiliations:** ^1^ Department of Physics, Kyushu University, Fukuoka, Japan; ^2^ Department of Chemical Engineering, Kyoto University, Kyoto, Japan

**Keywords:** cell motility, substrate adhesion, force-free, torque-free, traction force, mechano-chemical model, reaction-diffusion model, subcellular-element model

## Abstract

Constructing physical models of living cells and tissues is an extremely challenging task because of the high complexities of both intra- and intercellular processes. In addition, the force that a single cell generates vanishes in total due to the law of action and reaction. The typical mechanics of cell crawling involve periodic changes in the cell shape and in the adhesion characteristics of the cell to the substrate. However, the basic physical mechanisms by which a single cell coordinates these processes cooperatively to achieve autonomous migration are not yet well understood. To obtain a clearer grasp of how the intracellular force is converted to directional motion, we develop a basic mechanochemical model of a crawling cell based on subcellular elements with the focus on the dependence of the protrusion and contraction as well as the adhesion and de-adhesion processes on intracellular biochemical signals. By introducing reaction-diffusion equations that reproduce traveling waves of local chemical concentrations, we clarify that the chemical dependence of the cell-substrate adhesion dynamics determines the crawling direction and distance with one chemical wave. Finally, we also perform multipole analysis of the traction force to compare it with the experimental results. Our present work sheds light on how intracellular chemical reactions are converted to a directional cell migration under the force-free condition. Although the detailed mechanisms of actual cells are far more complicated than our simple model, we believe that this mechanochemical model is a good prototype for more realistic models.

## 1 Introduction

Autonomous migration is a fundamental function of biological cells, and it is of essential importance in many biological processes during development and homeostasis. A number of studies have been conducted to reveal how intracellular biochemical reactions break the symmetry of a cell so that it migrates directionally Swaney et al. (2010); Beta and Kruse (2017); Devreotes et al. (2017). In order to bridge the gap between intracellular chemical signals and spatial migration of cells, we have to consider how the force that a cell produces by itself is controlled by the intracellular chemical reactions and how it drives the cell.

From the viewpoint of mechanics, in general, objects that exhibit spontaneous motion are called active matter Lauga and Powers (2009); Ramaswamy (2010); Vicsek and Zafeiris (2012); Cates (2012); Marchetti et al. (2013); Bechinger et al. (2016). In contrast to objects passively driven by external forcing, active matter, including living cells, generates force in itself, which is characterized by a vanishing force monopole, i.e., vanishing simple sum of the force. This is because of the action-reaction law, which requires the existence of the counter force of internal force acting on another part of the active object with the same magnitude but in the opposite direction. Note that the internal force is generated *inside* the active object. Under this force-free condition, it is necessary to break symmetry to achieve spontaneous motion, such as directional motion. The same applies to the torque that active matter generates. That is, spontaneous rotation requires torque-free condition. For microorganisms that swim in a fluidic environment, the scallop theorem Purcell (1977) describes the importance of breaking reciprocality to achieve a net migration *via* internal cyclic motions. In particular, studies on a simple model of Purcell’s three-bead swimmer revealed that the phase shift between the two periodically stretching bonds can break time reversal symmetry to realize a net migration instead of reciprocating motion Najafi and Golestanian (2004); Günther and Kruse (2008).

**FIGURE 1:**
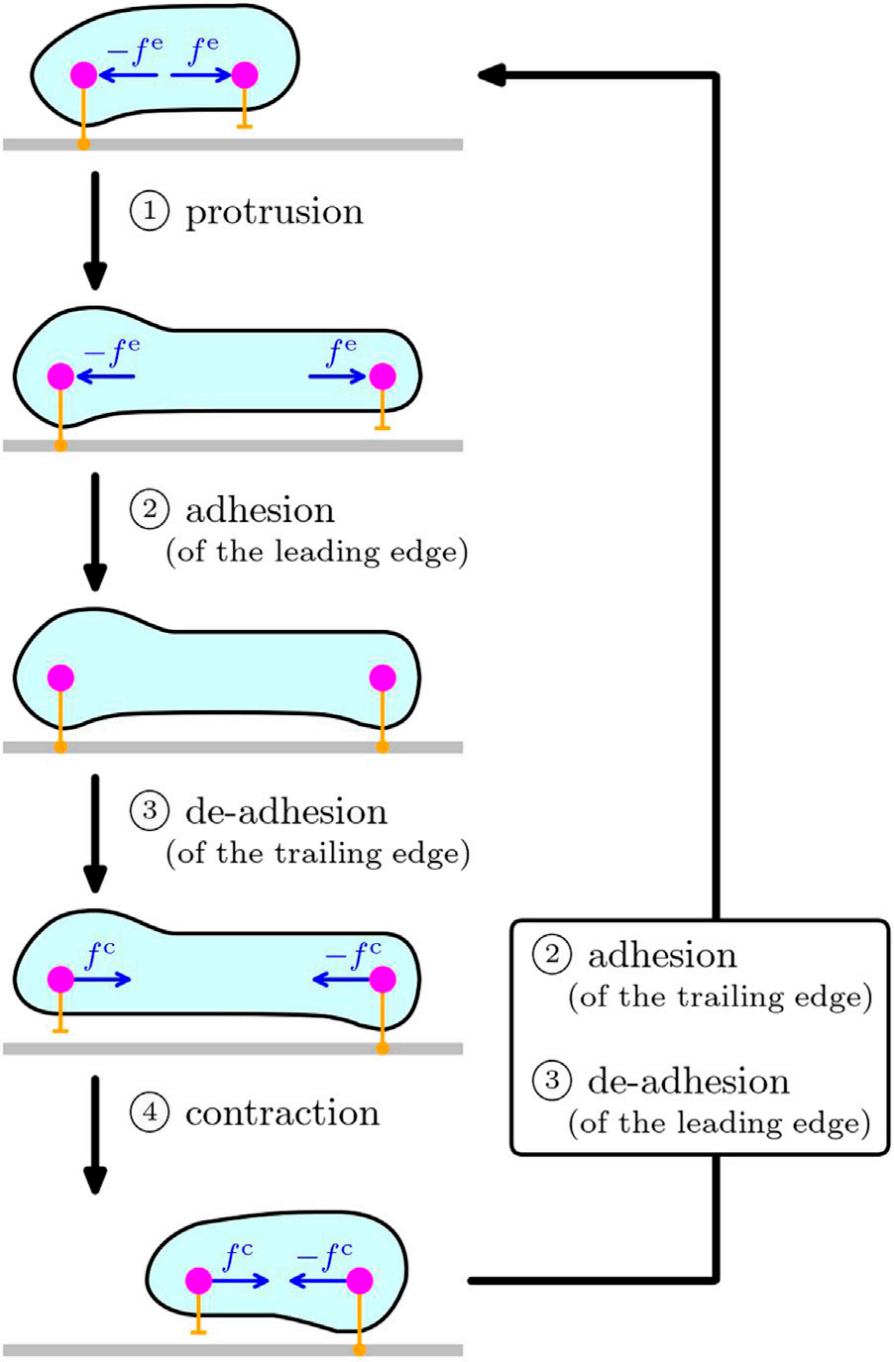
Sketch of the crawling cycle of a cell on substrate, consisting of the four processes: ① protrusion, ② adhesion, ③ de-adhesion, and ④ contraction. The cell depicted in cyan enclosed by membrane (black line) is crawling on the substrate (gray). The two magenta circles schematically represent the front and rear parts of the cell, and the orange bars symbolize the interaction with the substrate underneath. Protrusion is led by the extensile force *f* ^e^ on the leading edge generated by intracellular cytoskeleton, and thus, its counter force − *f* ^e^ acts on another part (i.e., the rear in the sketch) of the cell because of the law of action and reaction. In the same way, the contraction process is caused by the contractile force *f* ^c^ at the trailing edge due to the force generation of intracellular cytoskeleton, and its counter force − *f* ^c^ act on the front of the cell. The local interaction of the cell with the substrate underneath (orange bars) can change its characteristics between the adhered state and the de-adhered state. The processes ② and ③ in the box on the right represent the recovery of the substrate adhesion to close the crawling cycle.

In contrast to the locomotion of microswimmers that stir surrounding fluid to propel, adhesion to the substrate such as the extracellular matrix and other cells plays an important role in the locomotion of cells crawling on the substrates, on which the cells exert traction force. Typical mechanism of such cell crawling includes the following four processes, 1) protrusion, 2) adhesion to the substrate, 3) de-adhesion from the substrate, and 4) contraction, as sketched in Figure 1. This simple mechanism of crawling cycle is currently accepted in the biology community Ananthakrishnan and Ehrlicher, (2007). In this case, the force-free condition requires the existence of the counter force of the extensile and contractile force of cytoskeleton during the protrusion and contraction, that act on another part of the cell. See also the sketch in Figure 1. Note that the protrusion and contraction are induced by the dynamics of intracellular cytoskeleton Ananthakrishnan and Ehrlicher, (2007); Blanchoin et al. (2014); Tarama and Shibata (2022). In our previous study Tarama and Yamamoto (2018), we clarified the role of substrate adhesion by using a simple mechanical model in which a cell is described by two subcellular elements, that can switch their substrate friction periodically between the adhered stick state and the de-adhered slip state, and that are connected by a viscoelastic bond including an actuator that elongates and shrinks cyclically. By tuning the phase shifts between the actuator elongation and the substrate friction change of each subcellular element, we demonstrated that coupling between the cyclic intracellular force and the dynamic asymmetry of the substrate friction, has a great impact on the crawling distance and efficiency, as well as the crawling direction. By analogy to the Purcell’s three-bead swimmer model, this model cell can achieve a net crawling motion by adjusting the phase shift among the periodic stretching of cell body and the adhesion/de-adhesion dynamics at the front and rear of the cell. Several similar studies of simple inchwarm-style model have also shown that the regulation of substrate adhesion plays an important role in directional motion of cells on a substrate Kumar et al. (2008); Lopez et al. (2014); Mai and Camley (2020). We also note that many elaborate models have been introduced to explain various aspects of the cell crawling dynamics, including the cellular Potts model Nishimura et al. (2009); Niculescu et al. (2015), the continuous model Marchetti et al. (2013); Nier et al. (2016), the phase field model Ziebert and Aranson (2016); Shao et al. (2010); Taniguchi et al. (2013); Shi et al. (2013); Ziebert et al. (2012); Tjhung et al. (2015), and the particle-based model Newman (2007); Sandersius and Newman (2008); Basan et al. (2011); Zimmermann et al. (2016); Smeets et al. (2016).

In contrast to such mechanistic tuning of the phase shift, in real cells, these extension and contraction, as well as the adhesion/de-adhesion processes, are controlled by intracellular chemical reactions. Therefore, in this paper, we address the question how intracellular chemical reactions can drive the spatial migration of a crawling cell by controling periodic extension and contraction of cytoskeleton under the force- and torque-free conditions as well as the substrate adhesion dynamics. To this end, we develop a basic particle-based mechanical model for cell crawling based on the typical mechanism of cell crawling sketched in Figure 1, which is coupled to intracellular chemical reactions. By considering a cell crawling on a flat substrate, we describe a cell by a set of many subcellular elements connected by viscoelastic bonds Newman (2007) as a simple extension of our previous mechanical model to two dimensions Tarama and Yamamoto (2018). In addition, intracellular chemical reactions are represented by simple reaction-diffusion (RD) equations Murray (2002, 2003); Kuramoto (1984); Epstein and Pojman (1998); Pismen (2006), which trigger mechanical activities. We then couple the RD equations and mechanical models to achieve efficient migration. In particular, we focus on the time delay between the intracellular chemical reactions and cell mechanics, which corresponds to the ordering of the basic crawling processes.

## 2 Materials and methods

First, we introduce our mechanical model of a crawling cell and the RD equations representing intracellular chemical reactions. As for RD equations, we employ a previously introduced model for an intracellular chemical reaction observed experimentally. We then couple the mechanical model and the RD equations, which regulate the intracellular mechanical activities. In particular, we confine ourselves to studying possible couplings between the intracellular chemical and mechanical models.

### 2.1 Subcellular-element model

**FIGURE 2:**
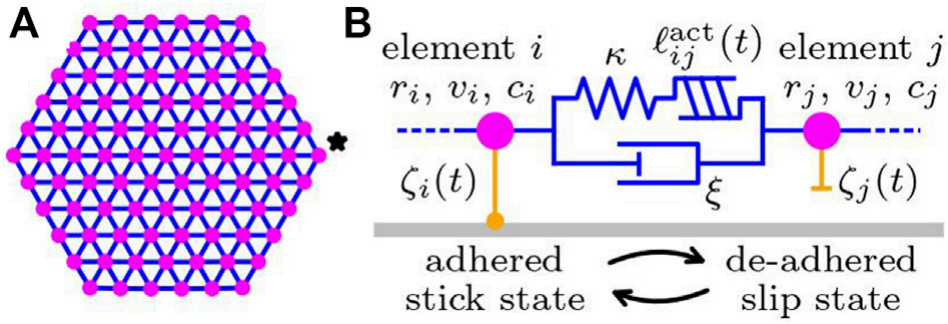
Sketch of the subcellular element model of a cell crawling on a substrate. **(A)** The cell is described by a set of subcellular elements (magenta circles) connected by viscoelastic bonds (blue lines). The shape of a cell at rest is assumed to be a perfect hexagonal lattice. The element indicated by the star is the activator element. **(B)** Details of the subcellular elements and the connecting viscoelastic bond. Each element possesses the chemical concentrations ***c***_*i*_. The actuator length $\ell_{ij}^{\text{act}}\left(t\right)$ and the substrate friction coefficient *ζ*_*i*_(*t*) change over time.

We describe a single cell by a set of subcellular elements Newman (2007) connected by Kelvin-Voigt type viscoelastic bonds, as schematically depicted in Figure 2. The elastic spring and damper of the Kelvin-Voigt bonds represent intracellular elasticity and dissipation. In addition, a linear actuator is included to the Kelvin-Voigt bond, which models expansion and contraction due to intracellular force generation by cytoskeleton. Each subcellular element interacts with the substrate underneath *via* the substrate friction proportional to the local velocity, where the substrate friction coefficient can change its characteristics between adhered stick state and de-adhered slip state. Since the typical size of a cell is on the order of 10 μm, the effect of inertia is negligible. Then, the force balance equation of element *i* is given by

$$\begin{array}{ll} & \zeta_{i}\left(t\right)v_{i}+\underset{j\in \Omega_{i}}{\sum }\xi \ell_{ij}\left(v_{i}-v_{j}\right)\\ & \;=\underset{j\in \Omega_{i}}{\sum }\frac{\kappa }{\ell_{ij}}{\hat{r}}_{ij}\left\{r_{ij}-\left(\ell_{ij}+\ell_{ij}^{\text{act}}\left(t\right)\right)\right\}+f_{i}^{\text{area}}, \end{array}$$ (1)

where ***v***_*i*_ is the velocity of the element *i* located at the position ***r***_*i*_. Here, the abbreviations *r* = | ***r*** | and $\hat{r}=r/r$ are used for the relative position ***r***_*ij*_ = ***r***_*j*_ − ***r***_*i*_. The summation is over the set of the elements Ω_*i*_ connected directly to the element *i* by the viscoelastic bonds. Note that in this paper we consider the case that the connection of the bonds between subcellular elements are permanent without breakup nor reconnection nor creation of new bonds for simplicity.

The first term on the left-hand side of Eq. 1 represents the substrate friction with coefficient *ζ*_*i*_(*t*), which changes over time due to intracellular activity. The second term represents intracellular dissipation with the rate *ξ*. The first term on the right-hand side represents intracellular elasticity with the elastic modulus *κ* and free length *ℓ*_*ij*_. Intracellular activity is also included in the actuator, which tends to elongate the connecting bond by changing the free length over time as $\ell_{ij}+\ell_{ij}^{\text{act}}\left(t\right)$ . Here, $\ell_{ij}^{\text{act}}\left(t\right)$ represents the actuator elongation, from which the force generated by the actuator is calculated as $f_{ij}^{\text{act}}\left(t\right)=-\kappa \ell_{ij}^{\text{act}}\left(t\right){\hat{r}}_{ij}/\ell_{ij}$ . We emphasize that the model Eq. 1 satisfies the force-free condition since the intracellular force acts symmetrically on the pair of subcellular elements. Namely, the sum of the intracellular force in Eq. 1 vanishes as

$$\underset{i}{\sum }f_{i}^{\text{int}}=0.$$ (2)

where

$$\begin{array}{ll} f_{i}^{\text{int}} & =-\underset{j\in \Omega_{i}}{\sum }\xi \ell_{ij}\left(v_{i}-v_{j}\right)\\ & \;+\underset{j\in \Omega_{i}}{\sum }\frac{\kappa }{\ell_{ij}}{\hat{r}}_{ij}\left\{r_{ij}-\left(\ell_{ij}+\ell_{ij}^{\text{act}}\left(t\right)\right)\right\}+f_{i}^{\text{area}} \end{array}$$ (3)

is the intracellular force acting on the element *i*.

The last term on the right-hand side of Eq. 1 prevents the collapse of the subcellular element network. It is given by $f_{i}^{\text{area}}=-\partial U^{\text{area}}/\partial r_{i}$ , where $U^{\text{area}}=\sum_{\left\langle i,j,k\right\rangle }\sigma /S_{\mathrm{ijk}}^{2}$ with *σ* = 10^–6^. This potential *U* ^area^ penalizes shrinking of the area of each triangle ⟨*i*, *j*, *k*⟩ formed by connected subcellular elements *i*, *j*, and *k*, which is defined by $S_{\mathrm{ijk}}=\left(r_{ij}\times r_{ik}\right)\cdot {\hat{e}}_{z}/2$ with ${\hat{e}}_{z}$ as the unit vector perpendicular to the 2D substrate.

We scale the system by *L*_0_ = 10 *μ*m for length and *T*_0_ = 1 min for time, which are physiologically relevant values for typical living cells Maeda et al. (2008); Tanimoto and Sano (2014). In addition, the scale of the force is set to *F*_0_ = 10 nN, which is on the order of the traction force that cells exert on the substrate. The typical values of the mechanical parameters of the model Eq. 1 are summarized in Table 1. We note that the dimensionless parameter constructed by the typical time scale, cell elasticity, and dissipation rate, that is related to the fluid-solid transition of cellular mechanics, is consistent with the typical value for a living cell summarized in Table 2.

**TABLE 1:** Mechanical parameters in the model and their values for the simulation of the hexagonal cell and the corresponding values in physical units. See also Table 2. The values in parentheses are for a large cell.

Model parameters	Simulation values	Typical values
Diameter of cell at rest, *L*_cell_	1 (1.4)	10 (14) *μ*m
Number of subcellular elements, *N*	91 (169)	
Number of bonds, *N*_bond_	240 (462)	
Bond length at rest, *ℓ*_*ij*_	$L_{\text{cell}}/\sqrt{N}$	≈1.05 (1.08) *μ*m
Total bond length at rest, *L*_bond_	$\sum_{\left\langle i,j\right\rangle }\ell_{ij}=N_{\text{bond}}L_{\text{cell}}/\sqrt{N}$	
Mean bond length, *ℓ*_0_	$L_{\text{bond}}/N_{\text{bond}}=L_{\text{cell}}/\sqrt{N}$	≈1.05 (1.08) *μ*m
Substrate friction coefficient		
in the slip state, *ζ*_slip_	$L_{\text{cell}}^{2}/N$	≈0.01 (0.006) nN (*μ*m)^−1^min
in the stick state, *ζ*_stick_	10*ζ*_slip_	≈0.1 (0.06) nN (*μ*m)^−1^min
Elastic modulus of each bond, *κ*	10	≈10 nN
Intracellular dissipation rate, *ξ*	$L_{\text{cell}}^{2}/L_{\text{bond}}$	≈0.01 (0.008) nN (*μ*m)^−1^min
Dimensionless parameter, *ωξ*_cell_/*k*_cell_	$2\pi \xi_{\text{cell}}/k_{\text{cell}}T_{0}=0.2\pi L_{\text{cell}}^{3}$	

**TABLE 2:** Mechanical parameter values for typical living cells and corresponding model parameters.

Mechanical parameters	Model	Living cells	References
Migration speed		$\approx 10\;\mu m{\left(min\right)}^{-1}$	Maeda et al. (2008)
Traction stress		≈100Pa	Tanimoto and Sano (2014)
Traction force		10 nN	(estimated)
Cell-substrate friction coefficient	*ζ*_cell_ = *ζ*_slip_ *N*	1 nN (*μ*m)^−1^min	(estimated)
Elastic modulus	$\kappa_{\text{cell}}=\kappa /L_{\text{cell}}^{2}$	10–100 Pa	Bausch et al. (1999); Micoulet et al. (2005)
Elastic constant	*k*_cell_ = *κ*/*L*_cell_	0.1–1 nN (*μ*m)^−1^	(estimated)
Dissipation rate	*ξ*_cell_ = *ξL*_bond_		
Fluid/solid transition time	*ξ*_cell_/*k*_cell_	tens of sec	Kasza et al. (2007)

### 2.2 Intracellular chemical reaction

In the model Eq. 1, the effects of the intracellular activities are included in the actuator elongation $\ell_{ij}^{\text{act}}\left(t\right)$ and the change in the substrate friction coefficient *ζ*_*i*_(*t*). The former represents the protrusion and contraction processes. The latter corresponds to the adhesion and de-adhesion of the cell to the underlying substrate. In actual cells, such cellular activities are caused by various intracellular chemical signals. However, it is not realistic to include all chemical components and their signaling pathways. Therefore, we model the intracellular chemical reactions by simple RD equations.

Taniguchi et al. gathered from their experimental observations of *Dictyostelium* cells that phosphatidylinositol (3,4,5)-trisphosphate (PIP3) promotes actin polymerization and protrusion of the cellular membrane, and therefore, they considered a signaling pathway around PIP3 including phosphatidylinositol (4,5)-bisphosphate (PIP2), PI3-kinase (PI3K), and phosphatase and tension homolog (PTEN). By eliminating the dynamics of PI3K and PTEN, they obtained the following set of RD equations Taniguchi et al. (2013):

$$\begin{array}{l} \frac{\partial U_{i}}{\partial t}=D_{U}\nabla^{2}U_{i}+G_{U}\left(U_{i},V_{i}\right),\\ \frac{\partial V_{i}}{\partial t}=D_{V}\nabla^{2}V_{i}+G_{V}\left(U_{i},V_{i}\right), \end{array}$$ (4)

where the reaction terms are defined as

$$\begin{array}{ll} & G_{U}\left(U,V\right)=-\frac{\alpha UV^{2}}{K_{K}+\langle V^{2}\rangle }+\frac{\beta UV}{K_{P}+\langle U\rangle }+S-\gamma U,\\ & G_{V}\left(U,V\right)=+\frac{\alpha UV^{2}}{K_{K}+\langle V^{2}\rangle }-\frac{\beta UV}{K_{P}+\langle U\rangle }-\mu V. \end{array}$$ (5)

The global couplings are given by

$$\langle U\rangle =\frac{1}{N}\underset{i}{\sum }U_{i},\;\langle V^{2}\rangle =\frac{1}{N}\underset{i}{\sum }V_{i}^{2},$$ (6)

where the summation is over all subcellular elements. Here, *U*_*i*_ and *V*_*i*_ represent the PIP2 and PIP3 concentrations for subcellular element *i*, respectively. The first and second terms in *G*_*U*_ represent the phosphorylation of PIP2 and the dephosphorylation of PIP3, respectively. The counterparts appear in the first two terms in *G*_*V*_. The third and fourth terms in *G*_*U*_ are constant production and degradation of PIP2. The last term of *G*_*V*_ represents a constant degradation of PIP3 Taniguchi et al. (2013). The global coupling terms originate from the conservation of the PI3K and PTEN concentrations.

To integrate Eq. 4, we calculate the Laplacian by using the moving particle semi-implicit (MPS) method Koshizuka et al. (1998). That is, for the chemical component *c*_*i*_ = {*U*_*i*_, *V*_*i*_}, the diffusion term is modeled by

$$D_{c}\nabla^{2}c_{i}=\frac{4D_{c}}{\lambda }\underset{j\neq i}{\sum }\frac{1}{n_{ij}}\left(c_{j}-c_{i}\right)w\left(r_{ij}\right),$$ (7)

where *n*_*ij*_ = (*n*_*i*_ + *n*_*j*_)/2, and *n*_*i*_ = *∑*_*j*≠*i*_ *w* (*r*_*ij*_) is the number of neighboring elements around element *i*. The weight function that we employed is defined by

$$w\left(r\right)=\left\{\begin{array}{cc} \frac{r_{e}}{r}-1\; & for\;0\leq r<r_{e}\\ 0\; & for\;r_{e}\leq r \end{array}\right.$$ (8)

where *r*_*e*_ is the cutoff length, which is set to 4*ℓ*_0_. The normalization factor *λ* is given by $r_{e}^{2}/6$ . By using this method, we checked whether the traveling and spiral waves are formed in the absence of the mechanical changes. To generate the wave, we add the stimulus (*δU*, *δV*) = (−*I*_excite_, + *I*_excite_) to Eq. 4 on the activator element, assuming that the phosphorylation of PIP2 to PIP3 is enhanced for this element.

**FIGURE 3:**
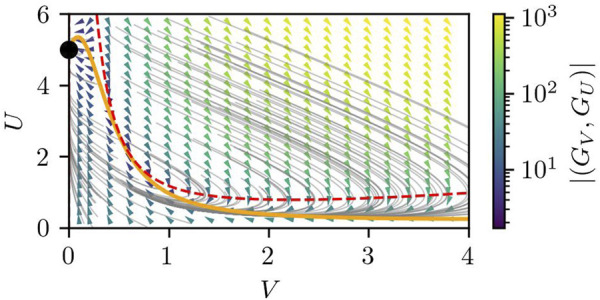
The nullcline and the flow field in the *U*-*V* space of Eqs 4, 5 under the assumption of uniform *U*(*r*) = *U* and *V*(*r*) = *V*. The orange solid line and the red dashed line correspond to the nullclines of *G*_*U*_(*U*, *V*) = 0 and *G*_*V*_(*U*, *V*) = 0, respectively, which correspond to *dU*/*dV* = 0 and *dV*/*dt* = 0. The gray lines show example trajectories starting from various (*U*, *V*), whereas the arrow heads and their color show the direction and magnitude of the flow (*G*_*V*_(*U*, *V*), *G*_*U*_(*U*, *V*)) at each phase point. The black dot represents the stable fixed point located at (*V*, *U*) = (0, *S*/*γ*).

The parameters of Eq. 4 that we used in this paper are summarized in Table 3. Here, the diffusion coefficient *D*_*U*_ = 0.48 corresponds to 0.8 μm^2^ (sec)^−1^, which is within the range of the experimentally-observed diffusion coefficient of proteins inside a cell near the membrane Golebiewska et al. (2008). We note that the choice of the other parameters is arbitrary, but what is of more importance than their absolute values is the nature of the RD equations, namely, the excitability, which is apparent from the nullclines and the flow field in the *U*–*V* space, as plotted in Figure 3.

**TABLE 3:** The parameter values of the RD equations in the simulation.

Model parameters	Values used in simulations
*D*_*U*_	0.48
*D*_*V*_	0.48
*α*	240
*β*	90
*K*_*K*_	5
*K*_*P*_	5
*S*	30
*γ*	6
*μ*	30

The important property of the RD equations, Eq. 4, is that they are of the Grey-Scott type Gray and Scott (1983, 1984). One of the advantages of the Grey-Scott model is that it can show either an excitable or a bistable nature depending on the parameters. Series of studies showed that the signaling pathway around PIP2-PIP3 reactions is excitable Nishikawa et al. (2014); Xiong et al. (2010); Huang et al. (2013); Devreotes et al. (2017); Gerisch et al. (2011, 2012); Gerhardt et al. (2014), which is also claimed in Taniguchi et al. (2013). Interestingly, similar RD equations were studied by Shao et al. Shao et al. (2010) in the context of cell crawling, where they assumed a bistable regime to reproduce the steady migration of keratocyte cells.

### 2.3 Mechanochemical coupling

To combine the cell mechanics, Eq. 1, and the RD equations, Eq. 4, we consider the coupling of the chemical concentrations to the actuator elongation $\ell_{ij}^{\text{act}}\left(t\right)$ and the substrate friction coefficient *ζ*_*i*_(*t*) individually.

#### 2.3.1 Actuator elongation

First, we introduce the coupling between the RD equations and the actuator elongation. Higher concentration of PIP3 enhances actin intensity Taniguchi et al. (2013), which we assume leads to actuator elongation. Then, we introduce the dependence of the actuator elongation on the PIP3 concentration, such that it elongates with PIP3 concentration as

$$\ell_{ij}^{\text{act}}\left(t\right)=\ell_{V}\tanh\left[aV_{ij}\left(t\right)\right],$$ (9)

where *V*_*ij*_(*t*) = (*V*_*i*_(*t*) + *V*_*j*_(*t*))/2 is the mean PIP3 concentration for the bond connecting the elements *i* and *j*. Although *V*_*i*_(*t*) is a positive quantity, its maximal value depends on the strength of the initial fluctuation because of the excitable nature of Eq. 4. Therefore, tanh is introduced on the right hand side of Eq. 9 to prevent an extremely large elongation. *a* is a constant denoting sensitivity, and *ℓ*_*V*_ is the magnitude of the elongation. Here, we set *a* = *π* and *ℓ*_*V*_ = *ℓ*_*ij*_.

#### 2.3.2 Substrate adhesion

**FIGURE 4:**
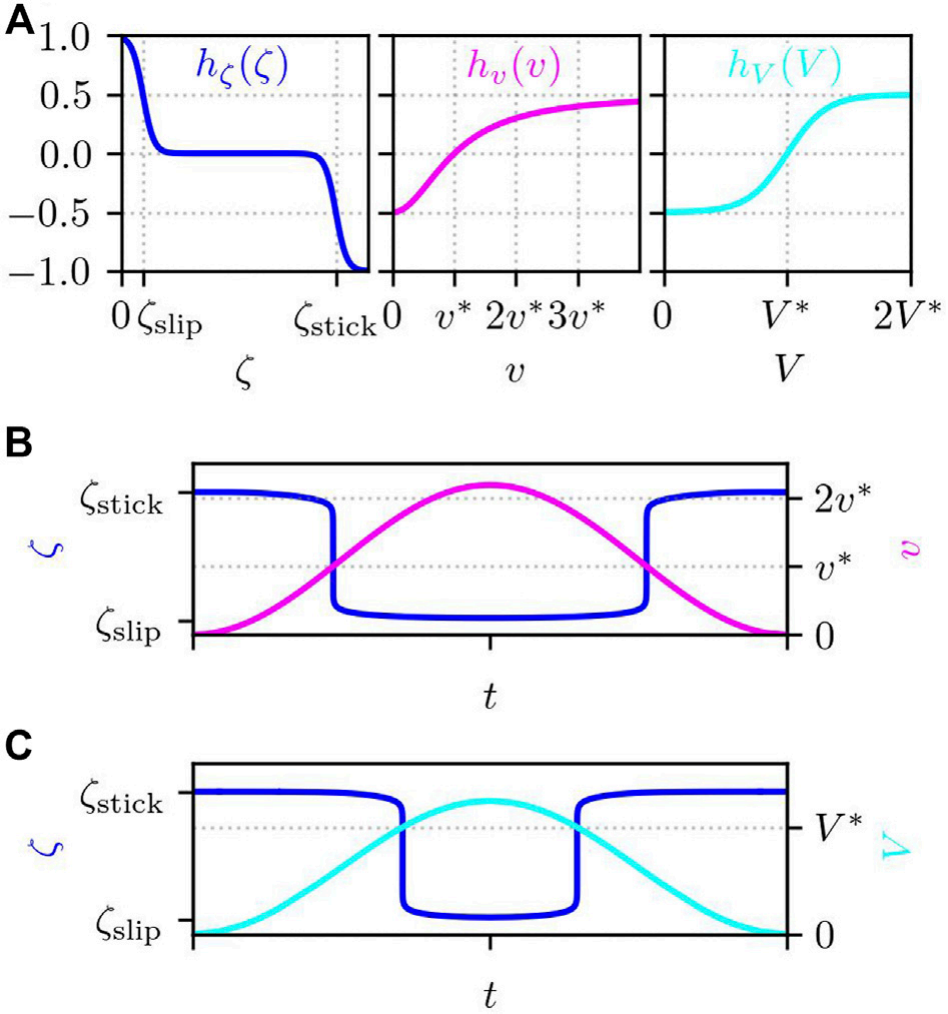
**(A)** Functional forms of *h*_*ζ*_(*ζ*), *h*_*v*_(*v*), and *h*_*V*_(*V*), which determine the substrate friction. Example trajectory of *ζ* that changes in time depending **(B)** only on the velocity *v* (*A*_*v*_ = 1) and **(C)** only on the PIP3 concentration *V* (*A*_*v*_ = 0). The parameters are set to the values given in Table 1.

Next, we consider the adhesion to the substrate underneath and the de-adhesion from it. We model the adhesion/de-adhesion processes by the transition of the substrate friction coefficient between the adhered stick state and the de-adhered slip state. Here, we consider the dependence of the substrate friction coefficient on both mechanical and chemical signals (see Figure 4A):

$$\tau_{\zeta }\frac{d\zeta_{i}}{dt}=h_{\zeta }\left(\zeta_{i}\right)-A_{v}h_{v}\left(v_{i}\right)-\left(1-A_{v}\right)h_{V}\left(V_{i}\right),$$ (10)

where *τ*_*ζ*_ is the time delay. *A*_*v*_ takes a value between 0 and 1, representing the ratio of the mechanical and chemical dependence of the stick-slip transition of the substrate friction.

The function *h*_*ζ*_(*ζ*) is defined by

$$h_{\zeta }\left(\zeta \right)=-\frac{1}{2}\tanh\left(\frac{\zeta -\zeta_{\text{stick}}}{\epsilon_{\zeta }}\right)-\frac{1}{2}\tanh\left(\frac{\zeta -\zeta_{\text{slip}}}{\epsilon_{\zeta }}\right).$$ (11)

Here, we assume a rapid transition between the adhered stick state *ζ*_stick_ and de-adhered slip state *ζ*_slip_, where the transition sharpness is given by *ϵ*_*ζ*_. Here, we set as *ϵ*_*ζ*_ = *ζ*_slip_/2. Note that the friction coefficient is smaller at the slip state than at the stick state, *ζ*_slip_ < *ζ*_*stick*_.

If we consider an artificial vesicle or droplet sitting on a substrate, its adhesion strength changes depending on the force acting on it Schwarz and Safran (2013). The term *h*_*v*_(*v*) in Eq. 10 represents this dependence of the cell adhesion to the substrate. Here, instead of the force acting on each subcellular element, we presume that the local velocity changes the adhesion strength through

$$h_{v}\left(v\right)=\frac{{\left(v/v^{*}\right)}^{2}}{1+{\left(v/v^{*}\right)}^{2}}-\frac{1}{2},$$ (12)

where v* is the threshold value. The subcellular element tends to adhere to the substrate (*ζ* = *ζ*_stick_) if the speed is smaller than the threshold value, i.e., *v* < v*, while the element slips on the substrate (*ζ* = *ζ*_slip_) if *v* > v*. We set the threshold value to v* = 1.

The formula of the stick-slip transition of the cell-substrate friction depending on the local velocity, i.e., Eq. 10 with *A*_*v*_ = 1, was introduced in Ref. Barnhart et al. (2015). As a result of the balance of the two functions, *h*_*ζ*_(*ζ*) and *h*_*v*_(*v*), the substrate friction switches between the stick and slip states with a sharp transition.

In addition to the mechanical dependence, the adhesion strength of a cell can change depending on its internal chemical conditions Schwarz and Safran (2013). Since the molecular details of cell adhesion are complicated, we assume here that it changes depending on the PIP3 concentration as the actuator elongation:

$$h_{V}\left(V\right)=\frac{1}{2}\tanh\left(\sigma_{V}\left(V-V^{*}\right)\right).$$ (13)

In Eq. 13, *σ*_*V*_ stands for the sensitivity, and V* is the threshold concentration. Due to this term, a large value of *V* prevents strong adhesion if *σ*_*V*_ > 0, while large *V* enhances the adhesion if *σ*_*V*_ < 0. However, we are not sure whether PIP3 enhances or diminishes adhesion. Therefore, in the next section, we numerically solve the time-evolution equations for both cases and see what will happen.

In Figures 4B,C, we show two example trajectories of *ζ* that changes depending only on the local velocity *v* (Figure 4B for *A*_*v*_ = 1) and on the PIP3 concentration *V* (Figure 4C for *A*_*v*_ = 0). Since both *h*_*v*_(*v*) and *h*_*V*_(*V*) take values between [ − 0.5, 0.5] and since 0 ≤ *A*_*v*_ ≤ 1, −0.5 ≤ *A*_*v*_ *h*_*v*_(*v*) + (1 − *A*_*v*_)*h*_*V*_(*V*) ≤ 0.5. Then, from Eq. 10, *ζ* takes a value between [*ζ*_slip_, *ζ*_stick_].

We note that the mechanical dependence of the stick-slip transition of the substrate friction is consistent with that of usual materials such as adhesive plastic tapes, which tends to de-adhere under strong mechanical force or velocity. In contrast, the chemical cue seems rather characteristic to biological cells, although to our knowledge the signaling pathway that regulates the substrate adhesion mediated by the focal adhesion is yet to be clarified.

### 2.4 Numerical analysis

To integrate the time-evolution Equations 1, 4 with Eqs 9, 10 numerically, we employ the Euler method with time increment *δt* = 10^–5^. We add the stimulus (*δU*, *δV*) = (−*I*_excite_, + *I*_excite_) to Eq. 4 on one of the subcellular elements, which we call the activator element. We set the activator element to the rightmost element denoted by the star in Figure 2A, except in the case of random motion, for which an activator element is chosen randomly every time *t* = 0.15. Since the traveling wave is generated by the stimuli on the activator element, we excite the activator element every *t* = 1.5 so that the wave travels to the other edge of the cell within that period, and all the subcellular elements relax back to the resting state when the next wave is generated. The intensity of the initial stimulus is set to *I*_excite_ = 0.75.

We note that most of the analyses in the following sections are performed by using a hexagonal cell shape, depicted in Figure 2. This is because it enables us to prepare a regular lattice of subcellular elements and, thus, is free from the complexity that the inhomogeneity of the position and connection of the subcellular elements may raise. In order to see the impact of this choice, we compare the results with the case of a circular cell shape in Sect. 3.5, which looks more relevant to realistic cells.

## 3 Results

### 3.1 Inhomogeneity of substrate adhesion

We start with considering the role of the inhomogeneity of the substrate adhesion on cell crawling. From Eq. 1, the centre-of-mass velocity is calculated as

$$V_{\text{cm}}=\frac{1}{N}\underset{i}{\sum }v_{i}=\frac{1}{N}\underset{i}{\sum }\frac{1}{\zeta_{i}\left(t\right)}f_{i}^{int},$$ (14)

where $f_{i}^{int}$ is defined by Eq. 3. If the substrate adhesion is homogeneous, *ζ*_*i*_(*t*) = *ζ*_0_(*t*) ≠ 0, Eq. 14 becomes

$$V_{\text{cm}}=\frac{1}{N\zeta_{0}\left(t\right)}\underset{i}{\sum }f_{i}^{\text{int}}=0.$$ (15)

Here, the force-free condition, Eq. 2, is used at the second equality. Note that the time dependence of the homogeneous substrate adhesion does not lead to a finite centre-of-mass velocity. Note also that the summation in Eq. 15 can be replaced by the integral over the cell in the continuum limit without loosing generality. This demonstrates that spatial translational motion is not achieved without symmetry breaking of substrate adhesion under the force-free condition.

### 3.2 Sinusoidal traveling chemical wave

The analysis of the two-element case, i.e., dumbbell model in our previous paper Tarama and Yamamoto (2018) showed that the coupling between the asymmetric substrate friction and the actuator elongation affects the crawling efficiency. In fact, there exists a reciprocating motion where a cell propels in one direction and moves in the opposite direction for the same distance for the rest of the period, resulting in no net migration. In this section, we test if this coupling also changes the crawling motion of a cell composed of many subcellular elements.

As a simple realization of inhomogeneous substrate adhesion, we consider a traveling harmonic wave of a single intracellular chemical component *c*(*t*):

$$c_{i}\left(t\right)=c_{0}\left(1-\cos\phi_{i}\left(t\right)\right),$$ (16)

where the phase changes as

$$\phi_{i}\left(t\right)=\omega t-q_{w}\left(x_{i}-x^{*}\right).$$ (17)

Here *c*_0_ is the maximum concentration, which is set to *c*_0_ = 0.5 so that 0 ≤ *c*_*i*_(*t*) ≤ 1. *ω* and *q*_*w*_ are the frequency and the wavenumber of the traveling wave, and x* is the *x* position of the activator element, from which the traveling wave occurs. The dependence of the actuator elongation on the intracellular chemical signal Eq. 9 is replaced by

$$\ell_{ij}^{\text{act}}\left(t\right)=\ell_{c}\frac{c_{i}\left(t\right)+c_{j}\left(t\right)}{2},$$ (18)

where *ℓ*_*c*_ represents the magnitude of the elongation. The dynamics of the substrate friction coefficient *ζ*_*i*_(*t*) is also replaced by a simple two-state function that switches between the adhered stick state and the de-adhered slip state:

$$\zeta_{i}\left(t\right)=\left\{\begin{array}{cc} \zeta_{\text{slip}}\; & if\;2m_{i}\pi <\phi_{i}\left(t\right)-\psi_{\zeta }\leq \left(2m_{i}+1\right)\pi \\ \zeta_{\text{stick}}\; & if\;\left(2m_{i}+1\right)\pi <\phi_{i}\left(t\right)-\psi_{\zeta }\leq 2\left(m_{i}+1\right)\pi \end{array}\right.$$ (19)

where *m*_*i*_ is an integer that satisfies 2*m*_*i*_ *π* < *ϕ*_*i*_(*t*) − *ψ*_*ζ*_ ≤ 2 (*m*_*i*_ + 1)*π*. *ψ*_*ζ*_ is a phase shift between the asymmetric substrate friction and the actuator elongation. Since these internal motions, i.e., the actuator elongation and the stick-slip transition of substrate friction, are perfectly cyclic, this phase shift controls the coupling between the asymmetric substrate friction and the actuator elongation.

We carried out numerical simulations of Eq. 1 together with Eqs 16–19 with the time increment *δt* = 10^–4^. In the simulation, we choose the element indicated by the star in Figure 2A as the activator element, and we set *q*_*w*_ > 0 so that the wave travels from right to left with the frequency *ω* = 2*π*. We let the actuator elongate twice as long as its equivalent length: *ℓ*_*c*_ = *ℓ*_*ij*_. For these parameters, we vary the wavenumber *q*_*w*_ and the phase shift *ψ*_*ζ*_ and measure the migration distance for one cycle.

**FIGURE 5:**
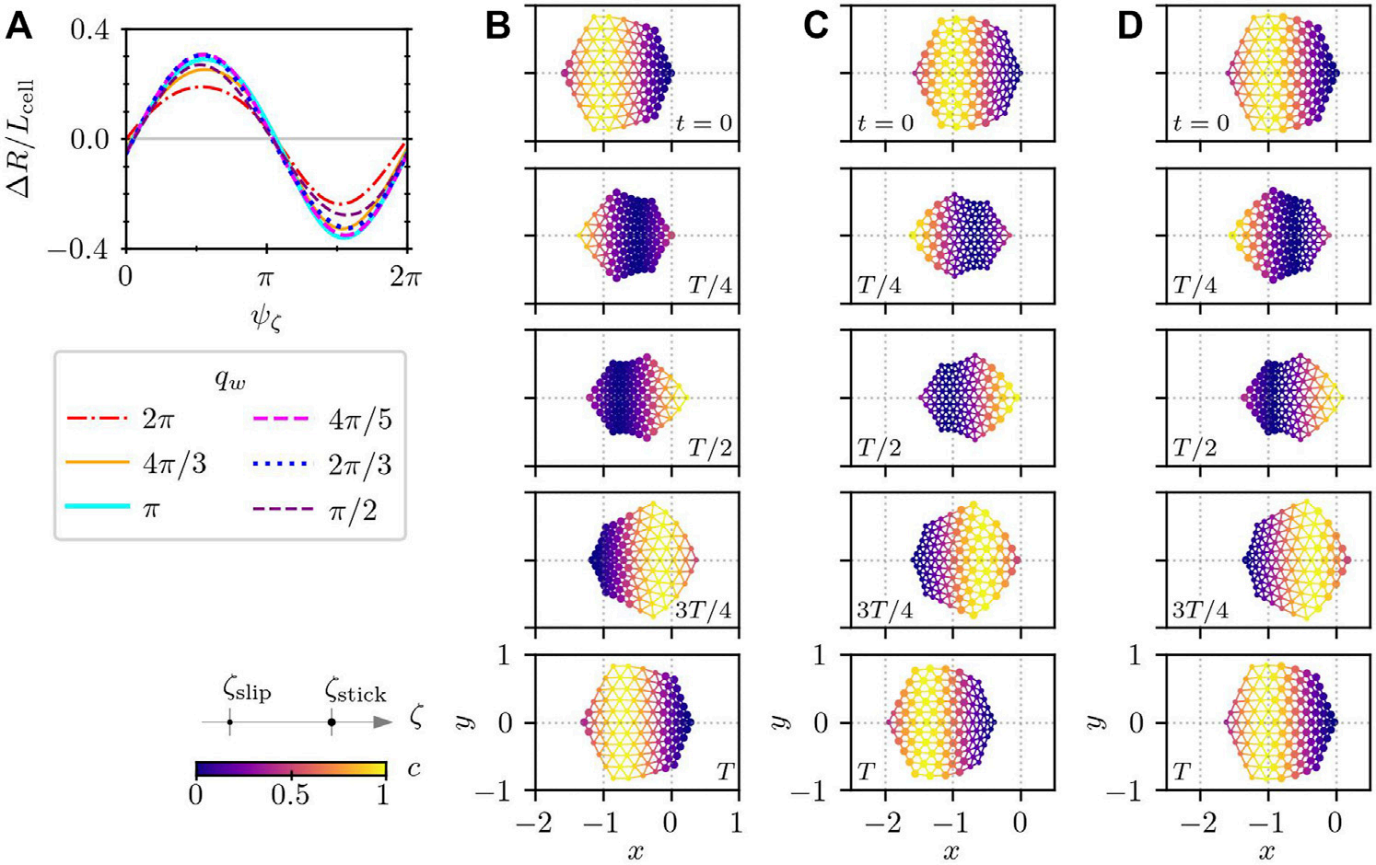
Cell crawling driven by a prescribed simple wave of intracellular chemical concentration, that is given in the form of a harmonic wave with the frequency *ω* = 2*π* and the wavelength *L*_*w*_ that travels from the rightmost subcellular elements to the left. **(A)** Migration distance for different values of the wavelength *L*_*w*_ and the phase shift *ψ*_*ζ*_. The displacement in one cycle is measured after several cycles of relaxation. The sign of Δ*R* distinguishes the direction of motion. The positive and negative signs of Δ*R* correspond to the rightward and leftward motion, respectively. The frequency of the traveling wave is set to *ω* = 2*π*. [**(B)**–**(D)**] Time series of crawling cells for *q*_*w*_ = *π*. **(B)** Rightward motion for *ψ*_*ζ*_ = 5*π*/9, **(C)** leftward motion for *ψ*_*ζ*_ = 14*π*/9, and **(D)** reciprocating motion for *ψ*_*ζ*_ = 4*π*/90. In panels **(B)**–**(D)**, the numbers show the time, and the color indicates the distribution of the intracellular chemical component *c*(*t*). The size of the subcellular elements represents the substrate friction; large and small elements correspond to the adhered stick state and the de-adhered slip state, respectively.

Figure 5A shows that the migration distance for one cycle Δ*R* alters depending on the phase shift *ψ*_*ζ*_. The sign of Δ*R* distinguishes the migration direction. Positive Δ*R* represents rightward motion, whereas negative Δ*R* corresponds to leftward motion. Since the wave of the intracellular chemical concentration *c*_*i*_ travels from rightmost subcellular element toward the left of the cell, the direction of the rightward cell migration is opposite to that of the traveling wave, as depicted in Figure 5B. Note that the cell shape at time *t* = 3*T*/4 looks like a flipped version of the cell at *t* = 0 in Figure 5B. This is because the phase of the intracellular chemical concentration in Eq. 17 is defined as a function of the distance with respect to the rightmost subcellular element and the length of the bonds connecting subcellular elements changes due to the actuator elongation. On the other hand, in the case of the leftward migration, the cell migrates in the same direction as that of the intracellular traveling wave of *c*_*i*_ as shown in Figure 5C. At the phase shift *ψ*_*ζ*_ where the migration direction switches from the rightward migration to the leftward motion, reciprocating motion appears, as depicted in Figure 5D. In reciprocating motion, the cell migrates in one direction (to the left in the case of Figure 5D) during some time of the period, and migrates in the other direction (to the right in Figure 5D) for the same distance during the rest of the period. Therefore, the reciprocating motion results in no net migration, and thus, Δ*R* = 0. Note that the asymmetry of substrate friction exists even in the cell undergoing the reciprocating motion. The migration distance Δ*R* also depends on the wavenumber *q*_*w*_. There is an optimal *q*_*w*_ around *q*_*w*_ = 4*π*/5 for which the cell can achieve the largest migration distance.

These results highlight that in addition to the substrate friction asymmetry, the spatial motion of a cell changes depending on the phase shift between the force generation and the formation of asymmetric adhesion, which corresponds to the time delay in Eq. 10 where the intracellular chemical reaction is controlled by reaction-diffusion equations.

### 3.3 Crawling by direct and retrograde waves

**FIGURE 6:**
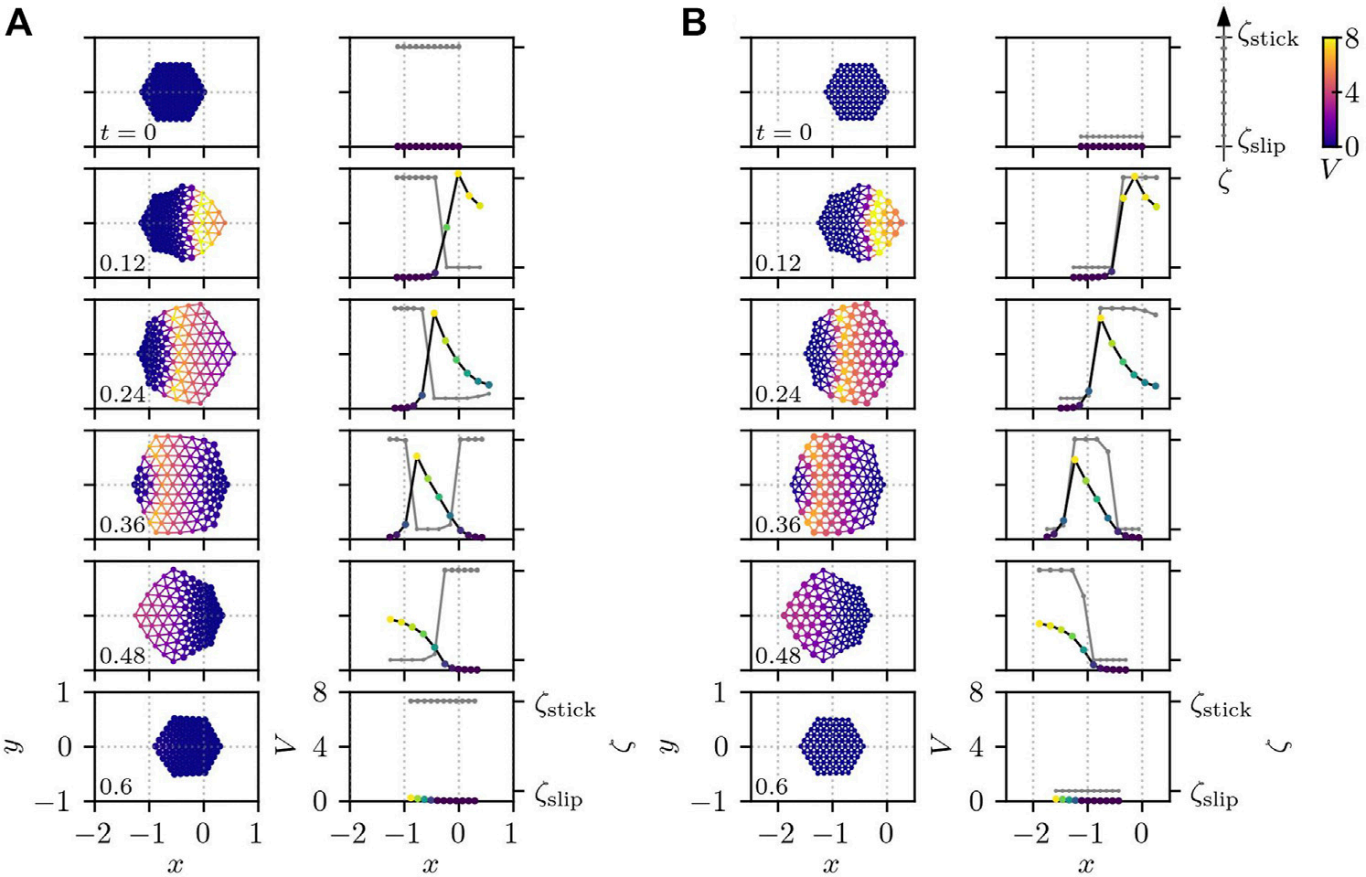
Cell crawling obtained from Eqs 1, 4 with Eqs 9, 10 for different signs of *σ*_*V*_: **(A)** *σ*_*V*_ = 2*π* and **(B)** *σ*_*V*_ = −2*π*. The cell for positive *σ*_*V*_ crawls in the opposite direction against the traveling chemical wave as shown in panel **(A)**, whereas, for negative *σ*_*V*_, it moves in the same direction as the wave as displayed in panel **(B)**. The other parameters are set to *A*_*v*_ = 0 and *τ*_*ζ*_ = 0.01. In both panels, the first column depicts two-dimensional snapshots, and the second column shows the corresponding values of *V*_*i*_ (by dots with color that also represents the value of *V*_*i*_, connected by black line) and *ζ*_*i*_ (in gray) along the cross-section at *y* = 0. The position of each subcellular element is plotted by a circle whose size and color indicate the value of *ζ*_*i*_ and *V*_*i*_, respectively. The color of the connecting bonds corresponds to *V*_*ij*_. The number in the bottom left corner of each subplot represents the time.

Now we consider the situation where the cell mechanics Eq. 1 is driven by intracellular chemical reaction Eq. 4. First, we study the effect of the sign of *σ*_*V*_, by numerically integrating Eqs 1, 4 with Eqs 9, 10 for both positive and negative *σ*_*V*_. Figure 6 depicts a time series of snapshots of a crawling cell for *σ*_*V*_ = 2*π* in Figure 6A and *σ*_*V*_ = −2*π* in Figure 6B, respectively. In both Figures 6A,B, the first column depicts two-dimensional snapshots, and the second column shows the corresponding values of *V*_*i*_ (by dots with color that also represents the value of *V*_*i*_, connected by black line) and *ζ*_*i*_ (in gray) along the cross-section at *y* = 0. We set the threshold in Eq. 13 to V* = 0.5 throughout this paper.

If *σ*_*V*_ is positive, the cell moves in the opposite direction to the PIP3 traveling wave, as shown in Figure 6A. Interestingly, however, if the sign of *σ*_*V*_ is negative, a qualitatively different result appears: namely, the cell starts to move in the same direction as the traveling wave, as displayed in Figure 6B. This difference in the direction of migration can be understood from the time-series of the values of *V*_*i*_ and *ζ*_*i*_ along the cross-section at *y* = 0 in Figure 6. In the case of positive *σ*_*V*_, the subcellular elements tend to be at the de-adhered slip state *ζ*_slip_ for high values of *V*_*i*_, when the actuator tries to elongate. This leads to the protrusion of cell edge around the origin of the traveling PIP3 wave, i.e., on the right side of the cell at the earlier time period (0 ≲ *t* ≲ 0.24), which is then adhered strongly to the substrate for the later time period (0.36 ≲ *t* ≲ 0.6) when *V*_*i*_ in this region has already returned close to the resting state (*V*_*i*_ ∼ 0). Therefore, in this case, protrusion around the origin of the wave leads to the cell migration in the opposite direction to the traveling wave. On the other hand, for negative *σ*_*V*_, the subcellular elements tend to adhere strongly to the substrate for higher *V*_*i*_. This makes the actuator elongation at the earlier time *t* ∼ 0.12 result in an almost symmetric deformation of the cell along the *x* axis at *y* = 0. At the later time 0.36 ≲ *t* ≲ 0.48, however the recovery of the PIP3 concentration *V*_*i*_ to the resting state makes the subcellular elements de-adhere from the substrate around the back of the PIP3 wave, which causes the actuator contraction to bring the right half of the cell towards the PIP3 wave traveling at the left half of the cell where the subcellular elements adheres strongly to the substrate. This effective contraction drives the cell in the same direction as the traveling wave in the case of negative *σ*_*V*_. Note that the actuator elongates for high values of the PIP3 concentration *V*_*i*_.

With respect to the migration direction, the traveling wave in the same direction is called the direct wave, while the one in the opposite direction is referred to as the retrograde wave. In this sense, the above crawling motion for positive *σ*_*V*_ in Figure 6A corresponds to the motion with the retrograde wave, and the one for *σ*_*V*_ < 0 in Figure 6B corresponds to the motion with the direct wave. We note that the crawling by the direct and retrograde waves are also reported in the model for adhesive locomotion of gastropods Iwamoto et al. (2014). Since the experiments in Ref. Taniguchi et al. (2013) show that cells move in the direction in which PIP3 concentration is increased and thus actin polymerization is enhanced, we set *σ*_*V*_ = 2*π* for the rest of this paper.

### 3.4 Mechanical vs chemical control of adhesion

Now, we study the effect of the mechanical and chemical dependence of substrate friction coefficient on cell crawling. In particular, we focus on the following two factors. One is the time delay *τ*_*ζ*_ controlling the phase shift of the change in the substrate friction characteristics with respect to the intracellular chemical wave and, thus, to the intracellular force generation due to the actuator elongation, which is also induced by the chemical wave. The other is the parameter *A*_*v*_, which gives the dependence ratio of the substrate friction *ζ* on the local velocity and on the intracellular chemical signal. *A*_*v*_ is varied between *A*_*v*_ = 0, corresponding to the case where the substrate friction is fully controlled by the intracellular chemical signals, and *A*_*v*_ = 1, where the characteristics of the substrate adhesion changes depending only on the mechanical load. To characterize the cell migration, we measure the migration distance Δ*R* of the cell in one cycle, i.e., with one traveling wave.

**FIGURE 7:**
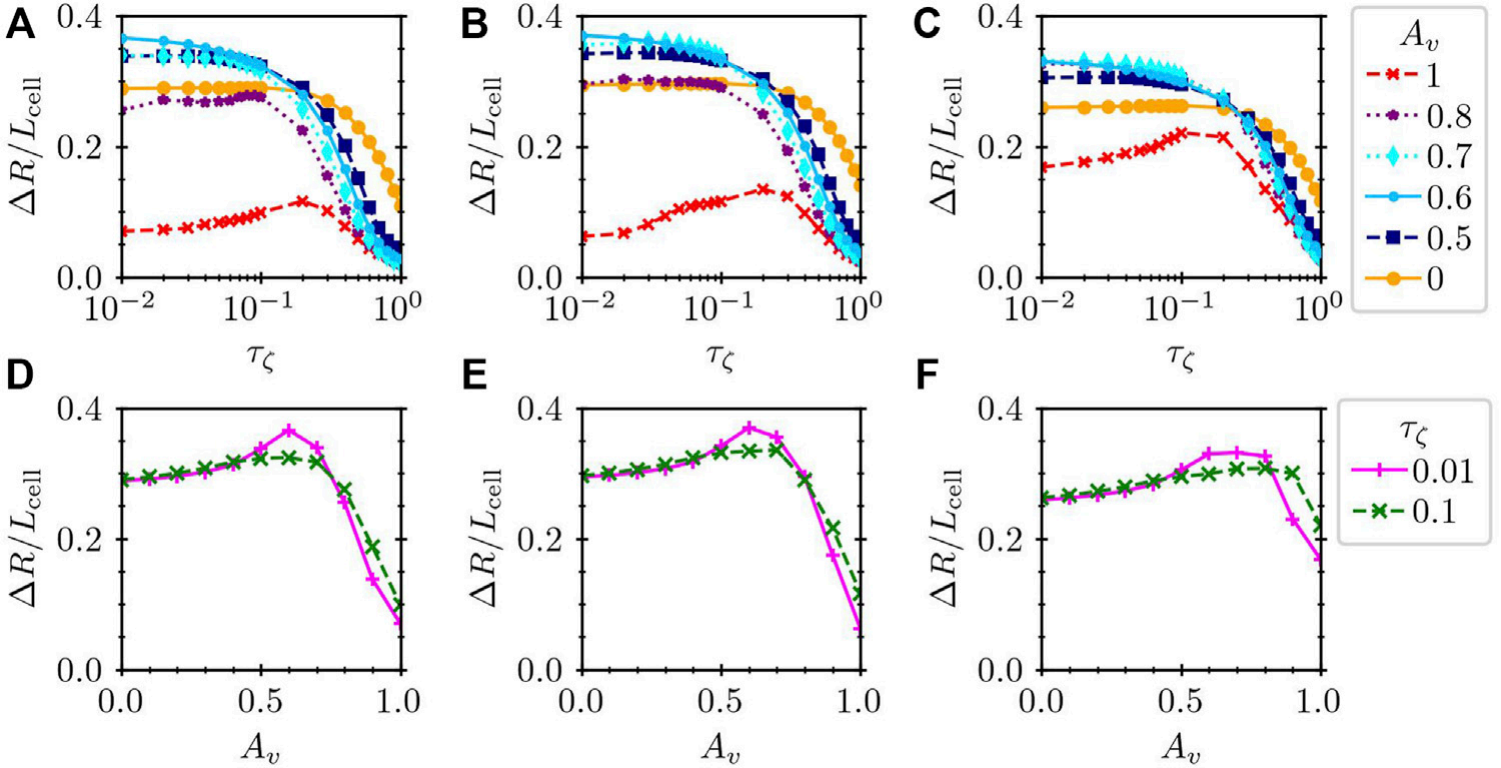
Normalized migration distance Δ*R*/*L*_cell_ **(A–C)** against the time delay *τ*_*ζ*_ and **(D–F)** against *A*_*v*_ for **(A,D)** a hexagonal cell (*L*_cell_ = 1), **(B,E)** a circular cell (*L*_cell_ = 1), and **(C,F)** a large hexagonal cell (*L*_cell_ = 1.4). In the panels **(A–C)**, the results for several values of *A*_*v*_ are plotted by different lines, whereas the dependence on *A*_*v*_ is shown for *τ*_*ζ*_ = 0.01 and 0.1 in the panels **(D–F)**.

In Figure 7A, we show the dependence on the time delay for different values of *A*_*v*_ as indicated in the legend. In all cases of *A*_*v*_, the dependence on the time delay appears strongest for large *τ*_*ζ*_. In this case, the response of the adhesion characteristics to the mechanical and chemical signals is so slow that the actuator elongation induced by the intracellular chemical wave of *V* is not well converted to a spatial migration of the cell. For a small *τ*_*ζ*_, the impact of the time delay becomes small. However, in the case of purely mechanically controlled substrate friction *A*_*v*_ = 1, the migration distance decreases for small *τ*_*ζ*_, and the migration is most efficient for the intermediate time delay around *τ*_*ζ*_ = 0.2. In addition, the migration distance Δ*R* is always much worse in the case of *A*_*V*_ = 1 than in the case of *A*_*v*_ = 0. When *A*_*v*_ = 1, the substrate friction characteristics changes depending only on the local speed and tends to be at the de-adhered slip state for high speed. Since this dependence does not distinguish the expanding and contracting processes, the substrate friction becomes the de-adhered slip state in both processes. Therefore, although the actuator elongation drives the cell front forward, the following actuator contraction moves it backward, resulting in a worse migration of the cell.

Interestingly, the mixing of the mechanical and chemical dependences of the substrate friction may result in larger values of Δ*R* than purely mechanical or chemical control. In order to highlight this point, we plot Δ*R* against *A*_*v*_ for *τ*_*ζ*_ = 0.01 and 0.1 in Figure 7D. The plot in Figure 7D clearly shows the maximum Δ*R* at the intermediate *A*_*v*_ ∼ 0.6. In Figures 7A,D, Δ*R*/*L*_cell_ reaches approximately 0.36 for *A*_*v*_ = 0.6 and *τ*_*ζ*_ = 0.01. This value is comparable to the measurement of a crawling *Dictyostelium* cell, which moves its body length in approximately two cycles Tanimoto and Sano (2014).

### 3.5 Impact of cellular shape

**FIGURE 8:**
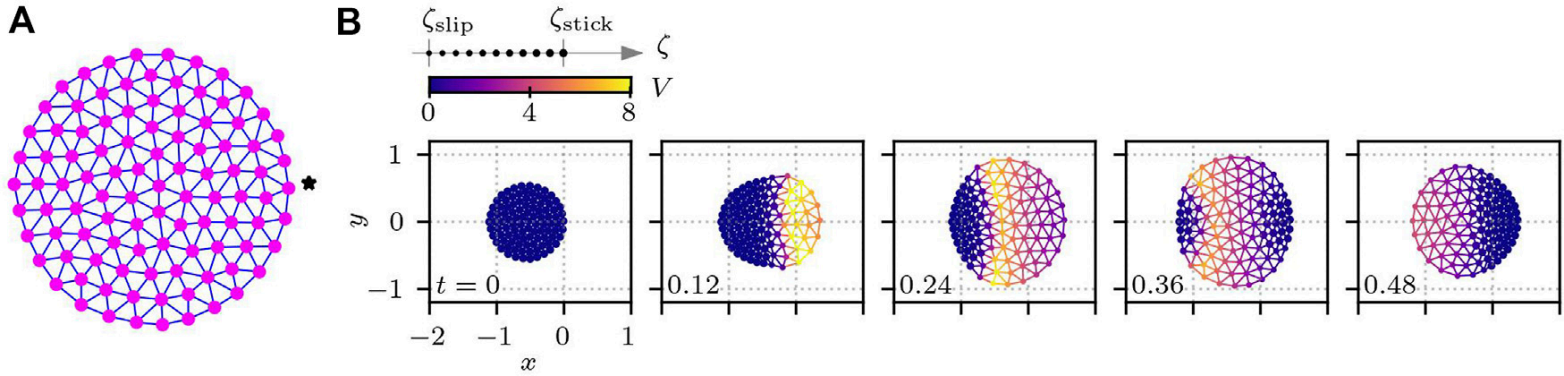
Cell with a circular shape. **(A)** Circular cell at rest. Subcellular elements plotted as magenta circles are connected by viscoelastic bonds indicated by blue lines. The details of the bond are the same as in Figure 2B. **(B)** Time series of crawling circular cells for *A*_*v*_ = 0.6, v* = 1, V* = 0.5, and *τ*_*ζ*_ = 0.01. In each subplot in panel **(B)**, the number in the bottom left corner shows the time, and the color indicates the value of *V*(*t*). The size of each subcellular element represents the value of the substrate friction coefficient.

Thus far, we have assumed a hexagonal cell shape, where the structure of the subcellular elements is given by a perfect hexagonal lattice when the cell is at rest. However, this structure does not describe real cells, which are instead circular or often of more complicated shapes. To elucidate the impact of the cell shape on the crawling motion, we prepare a cell of circular shape, where the subcellular elements are connected as depicted in Figure 8A. The length of the cell was set to *L*_cell_ = 1, and the total number of subcellular elements was set to *N* = 100. The total number of bonds connecting the subcellular elements was then *N*_bond_ = 267. We set *ℓ*_*ij*_ of each bond of the circular cell to the initial element distance, as shown in Figure 8A. Then, the mean bond length was calculated to be *ℓ*_0_ = 0.105724. The other parameters were kept the same as for hexagonal cells. Figure 8B shows an example of a time series of circular cell crawling obtained numerically from Eqs 1, 4 with Eqs 9, 10. Here, the activator element is set as the one denoted by the star in Figure 8A, which is then stimulated by (*δU*, *δV*) = (−*I*_excite_, + *I*_excite_) with *I*_excite_ = 0.75 every *t* = 1.5. We then measure the migration distance for different values of *A*_*v*_ and *τ*_*ζ*_. The results are plotted in Figures 7B,E, which are qualitatively the same as those of the hexagonal cell in Figures 7A,D. This means that the rest shape of the model cell has less impact on the crawling dynamics.

### 3.6 Impact of cell size

Now, we study the impact of the size of the cell. We prepare a hexagonal cell of size *L*_cell_ = 1.4, which is approximately twice the area of the previous hexagonal cells. We again measure the migration distance Δ*R*, which is normalized by the cell length *L*_cell_ to facilitate comparison with the previous results for *L*_cell_ = 1.

The results are summarized in Figures 7C,F. Qualitatively, the tendency is the same as that of the results in Figures 7A,D for *L*_cell_ = 1. Namely, the migration distance can be larger if the substrate friction depends both on the mechanical and chemical signals than if it depends on only either one of them.

### 3.7 Random excitation

In reality, cells change their migration direction over time. In our model, we can reproduce such motion by introducing intracellular stochasticity, which may originate from, e.g., the complexity of intracellular chemical reaction processes. Here, we randomly choose one element in every *t* = 0.15 and add to that element the stimulus (*δU*, *δV*) = (−*I*_excite_, + *I*_excite_) with intensity *I*_excite_ = 0.75.

**FIGURE 9:**
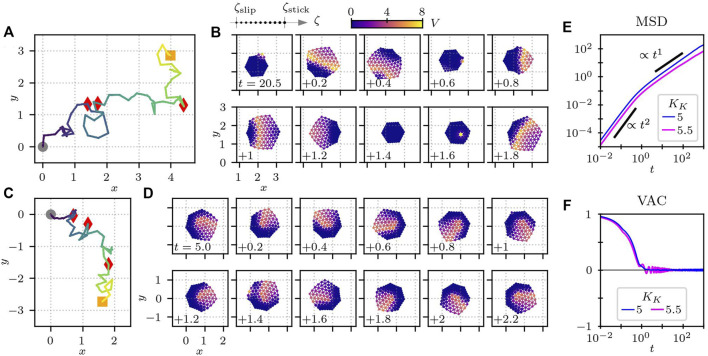
Cell crawling with randomly-chosen activator element for **(A,B)** *K*_*K*_ = 5 and **(C,D)** *K*_*K*_ = 5.5. **(E)** Mean-square displacement (MSD) and **(F)** velocity-autocorrelation function (VAC) for *K*_*K*_ = 5 and 5.5. In panels **(A)** and **(C)**, the trajectory of the center-of-mass position is plotted, where the gray circles indicate the initial position at *t* = 0, and the red triangles represent the position at every time interval of 10. The orange squares are the final position at time 40. **(B,D)** Time series of snapshots, **(B)** where the crawling direction of the cell changes and **(D)** where the cell undergoes a spinning motion. The numbers at the left bottom corners of the subplots in panels **(B)** and **(D)** show the time. The other parameters are the same as in Table 3.

The results are summarized in Figure 9. In Figures 9A,B, the parameter values are kept the same as in Figure 6A. Due to the stochasticity, however, the cell changes its migration direction frequently, as shown in the trajectory of the center-of-mass position in Figure 9A. From the snapshots of the cell in Figure 9B, we see that the migration direction depends on the position at which the chemical wave occurs. Because of the excitable nature of the RD equations, a stimulus on the element that has relaxed back to the resting state is more likely to be the origin of the next wave. Therefore, in principle, a new wave tends to originate from the elements that are near the origin of the previous wave.

To quantify the persistence of the migration direction, we measure the mean-square displacement (MSD), which is defined by $\left\langle {\left(X_{\text{cm}}\left(t_{0}+t\right)-X_{\text{cm}}\left(t_{0}\right)\right)}^{2}\right\rangle$ , where $X_{\text{cm}}$ is the center-of-mass position of the cell and ⟨*x* (*t*_0_)⟩ represents the time average of *x* (*t*_0_), i.e., the average of *x* (*t*_0_) over time *t*_0_. The results are displayed in Figure 9E. It shows a crossover from ballistic (∝ *t* ^2^) to diffusive regimes (∝ *t* ^1^) at around *t* ∼ 1. In fact, the velocity autocorrelation function (VAC) defined by $\left\langle {\tilde{V}}_{\text{cm}}\left(t_{0}+t\right)\cdot {\tilde{V}}_{\text{cm}}\left(t_{0}\right)\right\rangle$ for the direction of the center-of-mass velocity ${\tilde{V}}_{\text{cm}}{=V}_{\text{cm}}/{|V}_{\text{cm}}|$ , almost vanishes at this time scale, as shown in Figure 9F. If we compare this result with the experimental measurement in Ref. Li et al. (2008), which reported a persistent time of *t*_*p*_ = 8.8 ± 0.1 min, the value that our model reproduced is slightly smaller. We think this originates from the fact that our model cell lacks explicit polarity that memorizes the direction of migration.

If the parameter *K*_*K*_ in the reaction term of Eq. 5 is slightly increased from 5 to 5.5, the cell changes its migration direction more frequently, as depicted in Figure 9C. Depending on the random stimuli, the cell switches from directional motion to spinning motion as a spiral wave appears, as shown in Figure 9D. The spinning motion is rather stable, but the cell can also switch back to directional motion in response to a stimulus. Note that the RD equations maintain their excitable nature at this parameter value. In this case, the MSD shows a subdiffusive feature, where the MSD increases with the slop slightly smaller than *t* ^1^ for long time intervals, as shown in Figure 9E. This is probably because of the existence of the spinning motion that occurs from time to time. The spinning motion also leads to an oscillatory damping behaviour of the VAC, as plotted in Figure 9E.

### 3.8 Traction force multipoles

In many experiments, traction force that crawling cells exert on the substrate is measured Style et al. (2014) because of its fundamental importance for cell motility. The traction force results from the interaction between the cell and the substrate: $f_{i}^{\text{traction}}=\zeta_{i}\left(t\right)v_{i}$ . Since we are interested in cellular scale dynamics, we calculate the traction force multipoles, which was also measured experimentally Tanimoto and Sano (2014).

**FIGURE 10:**
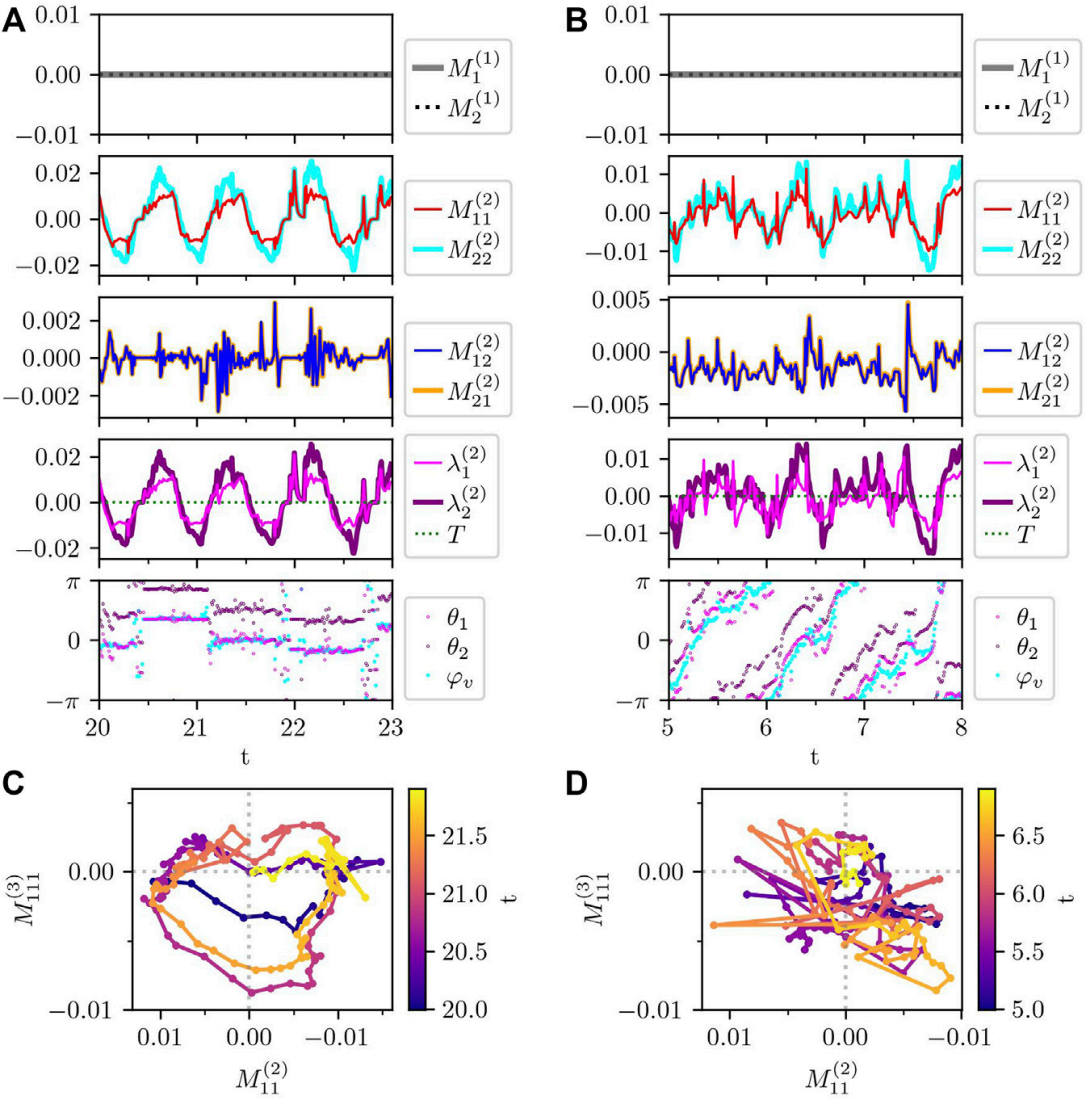
Traction force multipole of the randomly crawling cells in Figure 9. **(A,B)** Times series of each component of traction force monopole ( $M_{1}^{\left(1\right)}$ and $M_{2}^{\left(1\right)}$ ) and the diagonal ( $M_{11}^{\left(2\right)}$ and $M_{22}^{\left(2\right)}$ ) and off-diagonal components ( $M_{12}^{\left(2\right)}$ and $M_{21}^{\left(2\right)}$ ) of the traction force dipole, and the two eigenvalues of the force dipole tensor ( $\lambda_{1}^{\left(2\right)}$ and $\lambda_{2}^{\left(2\right)}$ ) and the torque (*T*), as well as the angle of the velocity (*φ*_*v*_) and the two eigenvectors of the traction force dipole (*θ*_1_ and *θ*_2_). **(C,D)** Time evolution of traction force dipole $M_{11}^{\left(2\right)}$ and quadrupole $M_{111}^{\left(3\right)}$ . The color indicates the time. The data in panels **(A,C)** are for the cell in Figure 9A, whereas those in panels **(B,D)** are for the cell in Figure 9B. Note that the axis of the $M_{11}^{\left(2\right)}$ is inverted, to match the plot in Ref. Tanimoto and Sano (2014) in panels **(C,D)**.

First, the traction force monopole is defined by

$$M_{\alpha }^{\left(1\right)}=\underset{i}{\sum }f_{i,\alpha }^{\text{traction}},$$ (20)

where *i* represents the subcellular element *i* and the summation runs over the entire cell. The subscript *α* indicates spatial component *α* = 1, 2, corresponding to *x* and *y* coordinates. Here, to compare with the experimental results in Ref. Tanimoto and Sano (2014), the traction force multipoles are calculated in the comoving coordinate on the cell, and thus, *α* = 1 and 2 represent the components parallel and perpendicular to the instantaneous center-of-mass velocity, respectively. In the numerical simulation of the model crawling cell, the traction force monopole is equal to 0, as shown in Figure 10, which is consistent with the experimental result Tanimoto and Sano (2014). This is readily understood from the force balance equation, Eq. 1, and the force-free condition, Eq. 2. That is, the traction force monopole vanishes because of the fact that the inertia is negligibly small for crawling cells on top of the force-free condition. Given that the force-free condition is a requirement for the intracellular force generation, we can understand that the vanishing force monopole obtained from the analysis of the experiment Tanimoto and Sano (2014) indicates the fact that the inertia is negligibly small for the crawling cell.

The next lowest mode is the traction force dipole, the element of which is defined by

$$M_{\alpha \beta }^{\left(2\right)}=\underset{i}{\sum }r_{i,\alpha }f_{i,\beta }^{\text{traction}}.$$ (21)

On the one hand, from Figures 10A,B, the diagonal components of the traction force dipole are oscillating around 0. Here, note that the positive and negative force dipoles represent the extensile and contractile force dipoles, respectively. In the experiment Tanimoto and Sano (2014), only the contractile traction force dipole was observed, which our results fail to reproduce. The reason why our model does not reproduce the contractile force dipole is not clear yet. One possibility is that the friction coefficient of the protrusion process may be different from that of the contraction process, which are set to the same in our current model.

On the other hand, the off-diagonal components $M_{12}^{\left(2\right)}$ and $M_{21}^{\left(2\right)}$ take the same values, indicating that the traction force dipole is symmetric, although such symmetry is not presumed in its definition, Eq. 21. Such symmetric property of the traction force dipole was also obtained in the experiment Tanimoto and Sano (2014).

Now, we consider the meaning of the symmetric property of the traction force dipole that is obtained in our simulation as well as in the experiment. Actually, this symmetric property of the traction force dipole indicates the torque-free nature of the cell. Here, the torque is defined by

$$T=\underset{i}{\sum }r_{i}\times f_{i}^{\text{traction}},$$ (22)

which, in a two dimensional space, becomes

$$T=\underset{i}{\sum }\left(r_{i,1}f_{i,2}^{\text{traction}}-r_{i,2}f_{i,1}^{\text{traction}}\right)=M_{12}^{\left(2\right)}-M_{21}^{\left(2\right)}$$ (23)

by using Eq. 21. Note that, if one describes the force dipole tensor with its invariants as in the fourth panels in Figures 10A,B, the torque appears as the imaginary part of the eigenvalues. Here, the trajectories of the two eigenvalues *λ*_1_ and *λ*_2_ of the traction force dipole look identical to those of $M_{11}^{\left(2\right)}$ and $M_{22}^{\left(2\right)}$ , in particular in Figure 10A. This is because the direction *θ*_1_ of the eigenvector of *M* ^(2)^ is basically in the same direction as the center-of-mass velocity (*φ*_*v*_) as shown in the last panel of Figure 10A. Here the eigenvalue *λ*_1_ is selected such that the direction of the corresponding eigenvector (*θ*_1_) is closer to *φ*_*v*_ than that of the other eigenvalue *λ*_2_. We emphasize that the torque-free property of the traction force is satisfied even by the spinning cell, as plotted in Figure 10B. In this case, the deviation of the angle *θ*_1_ of the eigenvector corresponding to *λ*_1_ from the angle *φ*_*v*_ of the center-of-mass velocity is larger as plotted in Figure 10B, resulting in the larger deviation of the trajectories of *λ*_1_ and *λ*_2_ from those of $M_{11}^{\left(2\right)}$ and $M_{22}^{\left(2\right)}$ .

Finally, in Figure 10C, we plot the time evolution of the traction force dipole $M_{11}^{\left(2\right)}$ and the traction force quadrupole $M_{111}^{\left(3\right)}$ , which is compared with the measurement in the experiment Tanimoto and Sano (2014). Here the traction force quadrupole is defined by

$$M_{\alpha \beta \gamma }^{\left(3\right)}=\underset{i}{\sum }r_{i,\alpha }r_{i,\beta }f_{i,\gamma }^{\text{traction}}.$$ (24)

Interestingly, the trajectory in $M_{11}^{\left(2\right)}$ - $M_{111}^{\left(3\right)}$ space shows a counterclockwise rotation, which is qualitatively consistent with the experimental results Tanimoto and Sano (2014). For the spinning cell, this counter-clockwise rotation is not clear in Figure 10D because it shows more rapid change in the magnitude of the traction force multipoles. Note that in the corresponding plot in Ref. Tanimoto and Sano (2014) the axis of the force dipole is inverted to highlight the magnitude of contraction. We followed this in Figures 10C,D where the axis of $M_{11}^{\left(2\right)}$ is inverted, to make the comparison easy.

## 4 Summary and discussion

To summarize, we investigate how intracellular chemical reactions can drive the spatial migration of a crawling cell by controling periodic extension and contraction of cytoskeleton under the force- and torque-free conditions as well as the substrate adhesion dynamics. To this end, we constructed a mechanochemical model of a cell crawling on a substrate based on the typical mechanism of cell crawling sketched in Figure 1. The mechanical part is described by a subcellular-element model, where we extend our previous model Tarama and Yamamoto (2018), and the chemical part is described by RD equations proposed by Taniguchi et al. (2013). To combine them, we introduce two mechanical activities. One is the actuator elongation, which depends on the intracellular chemical concentration. The other is the substrate friction, which shows a sharp transition between the adhered stick state and the de-adhered slip state depending on the intracellular chemical concentration in addition to the local velocity. We also introduce a time delay of the substrate friction change. Although we assumed a simple function form of the substrate friction dynamics and its dependence on the local velocity and intracellular chemical concentration, we believe that this time delay can be measured experimentally.

By using this model, we demonstrated that the substrate adhesion dynamics affect how the intracellular force leads to crawling motion under the force- and torque-free conditions. Both the substrate adhesion and intracellular force generation are controlled by the traveling wave of intracellular chemical reactions on the cellular scale. Depending on the sign of the sensitivity of the substrate friction coefficient to the intracellular chemical concentration, the model cell exhibited crawling with the retrograde flow or with the direct flow. In the former case, our model showed that there is an optimum time delay and that the combined effect of the mechanical and chemical signals on the substrate friction coefficient can increase the migration distance. We also investigated the impact of the cell shape and the cell size, which led to qualitatively the same results. In addition, by introducing stochasticity in the RD equations, the cell changes its migration direction and even switches its dynamical mode from translational motion to spinning motion. Further, we performed multipole analysis of the substrate traction force, which was qualitatively consistent with the experimental results except the contractile nature of the traction force dipole.

The persistence time of the cell crawling in our current model is slightly smaller than the experimental observation Li et al. (2008). The origin of the persistence in our current model is the excitable nature of the RD equations. That is, the intracellular chemical wave is triggered by a stimulus on the elements that is close to the resting state. Therefore, a new wave tends to occur from the region close to the origin of the previous wave, where the chemical signals have more chance to relax back to the resting state than the other part of the cell. This explains why our model cell cannot be persistent over too many wave cycles. Real cells, however, often establish a rather stable polarity, which allows the cells to migrate more persistently. This polarity can be modelled by bistable chemical reactions. In fact, recent experimental studies shed light on the coupling of the excitable chemical signaling pathways and the bistable chemical reactions, representing polarity Fukushima et al. (2019); Flemming et al. (2020). Therefore, it is important to include polarity to the model and study how it changes the relation among the intracellular chemical reactions and the force- and torque-free intracellular mechanics and the spatial migration of cells to realize a persistent crawling migration. We will come to this point in near future.

Finally, we discuss other possible extensions of our current model.

*Contraction process*: In our current model, the protrusion and contraction processes are both modeled by the actuator elongation, $\ell_{ij}^{\text{act}}\left(t\right)$ , of the bond connecting two subcellular elements. The two processes are distinguished by the sign of $\ell_{ij}^{\text{act}}\left(t\right)$ . In this paper, however, we consider only the protrusion process, i.e., $\ell_{ij}^{\text{act}}\left(t\right)\geq 0$ , which is related to the PIP3 concentration. One reason of this is that the chemical reactions that regulate contraction process are not well understood yet. Therefore, in principle, we can also consider the contraction process by introducing dependence on relevant chemical concentrations.

*Adhesion dynamics*: The cell adhesion is simply modeled by the switching of the substrate friction coefficient of each subcellular element in our model. However, in real cells, adhesion is mediated by adhesion molecules, which can diffuse and form focal adhesions. To represent these processes of adhesion molecules, we should extend our current model to include more detailed dynamics of the concentration of adhesion molecules and their diffusion to other subcellular elements. Then, we can discuss realistic structures such as the footstep-like focal adhesion observed for *Dictyostelium* cells Tanimoto and Sano (2014).

*Shape deformation*: Our model cell shows a lateral expansion with respect to the crawling direction, as shown in Figure 6A. However, real cells, e.g., *Dictyostelium* cells, tend to elongate in the direction of motion Maeda et al. (2008); Bosgraaf and Van Haastert (2009). One possible reason that our current model fails to reproduce this elongated shape is that actuator elongation depends only on the absolute value of *V*_*i*_. Therefore, this inconsistency may be resolved by, e.g., introducing dependence on the gradient of *V*_*i*_.In addition, the network of the subcellular elements is permanent in our current model. However, it is interesting to see what will happen by including the reconnection of the subcellular elements that evolves with the dynamics of the subcellular elements. With this extension, we expect more complicated deformation of the cell shape, which may look more realistic.

*Three dimensions*: In this paper, we modeled a cell as a two-dimensional network of viscoelastic springs by assuming crawling on a flat substrate. In reality, however, cells are three dimensional object. The extension of our current model to three dimensions is straightforward.

In conclusion, the modeling of crawling cells is still a challenging task because of the complexity in intracellular processes and of the variety of the cell crawling mechanism. By focusing on the mechanism how intracellular chemical reactions control the periodic extension and contraction of intracellular cytoskeleton to achieve a net directional migration under the force- and torque-free conditions (Figure 1), we demonstrated that the symmetry breaking due to inhomogeneous substrate adhesion and its time delay are of fundamental importance. In real cells, there are a huge variety of intracellular chemical reactions, and often they are related to each other. Nevertheless, we believe that this study can provide a basic framework to understand the synergetic mechanism of intracellular chemical reactions to realize effective migration of complex real cells by controlling the extension and contraction of cytoskeleton under the force- and torque-free conditions.

## Data Availability

The raw data supporting the conclusions of this article will be made available by the authors, without undue reservation.
